# Supplementary material for: Effects of short-chain fatty acid-butyrate supplementation on expression of circadian-clock genes, sleep quality, and inflammation in patients with active ulcerative colitis: a double-blind randomized controlled trial
Source: Lipids Health Dis. 2024 Jul 13;23:216. doi: 10.1186/s12944-024-02203-z (PMC11245831; doi:10.1186/s12944-024-02203-z)
Supplement: Supplementary file 1 — Supplementary Material 1 [file 12944_2024_2203_MOESM1_ESM.pdf]

Short IBDQ-9

The questions in this section are related to some gastrointestinal symptoms and the patient's quality of life in recent weeks:

1. How frequent have your bowel movements been during the last 2 wk?

- Bowel movements as or more frequent than they have ever been. 1. ☐
- Extremely frequent. 2. ☐
- Very frequent. 3. ☐
- Moderate increase in frequency of bowel movements. 4. ☐
- Some increase in frequency of bowel movements. 5. ☐
- Slight increase in frequency of bowel movements. 6. ☐
- Normal, no increase in frequency of bowel movements. 7. ☐

2. How often has the feeling of fatigue or of being tired and worn out been a problem for you during the last 2 wk?

- All of the time. 1. ☐
- Most of the time. 2. ☐
- A good bit of the time. 3. ☐
- Some of the time. 4. ☐
- A little bit of the time. 5. ☐
- Hardly any of the time. 6. ☐
- None of the time. 7. ☐

3. How much energy have you had during the last 2 wk?

- Most energy felt in years 1. ☐
- Much more than usual 2. ☐

- Slight increase 3. ☐

- About the same 4. ☐

- Slight decrease 5. ☐

- Much less than usual. 6. ☐

- Wiped out 7. ☐

4. How often during the last 2 wk have you had to delay or cancel a social engagement because of your bowel problem?

- All of the time. 1. ☐

- Most of the time. 2. ☐

- A good bit of the time. 3. ☐

- Some of the time. 4. ☐

- A little bit of the time. 5. ☐

- Hardly any of the time. 6. ☐

- None of the time. 7. ☐

5. How often during the last 2 wk have you been troubled by cramps in your abdomen?

- All of the time. 1. ☐

- Most of time. 2. ☐

- A good bit of the time. 3. ☐

- Some of the time. 4. ☐

- A little bit of the time. 5. ☐

- Hardly any of the time. 6. ☐

- None of the time. 7. ☐

6. How often during the last 2 wk have you felt generally unwell?

- All of the time. 1. ☐
- Most of the time. 2. ☐
- A good bit of the time. 3. ☐
- Some of the time. 4. ☐
- A little bit of the time. 5. ☐
- Hardly any of the time. 6. ☐
- None of the time. 7. ☐

7. Overall, in the last 2 wk, how much of a problem have you had with passing a large amount of gas?

- A major problem 1. ☐
- A big problem 2. ☐
- A significant problem 3. ☐
- Some trouble 4. ☐
- A little trouble 5. ☐
- Hardly any trouble 6. ☐
- No trouble 7. ☐

8. How much of the time during the last 2 wk have you been troubled by feeling nauseated or sick to your stomach?

- All of the time. 1. ☐
- Most of the time. 2. ☐
- A good bit of the time. 3. ☐
- Some of the time. 4. ☐
- A little bit of the time. 5. ☐

- Hardly any of the time. 6. ☐

- None of the time. 7. ☐

9. How satisfied, happy, or pleased have you been with your personal life during the last 2 wk?

- Very dissatisfied, unhappy mostly 1. ☐

- Generally dissatisfied, unhappy 2. ☐

- Somewhat dissatisfied, unhappy 3. ☐

- Generally satisfied, pleased 4. ☐

- Satisfied most of the time, happy 5. ☐

- Very satisfied most of the time, happy 6. ☐

- Extremely satisfied, could not have been more happy or pleased 7. ☐
