# Supplementary material for: Effects of short-chain fatty acid-butyrate supplementation on expression of circadian-clock genes, sleep quality, and inflammation in patients with active ulcerative colitis: a double-blind randomized controlled trial
Source: Lipids Health Dis. 2024 Jul 13;23:216. doi: 10.1186/s12944-024-02203-z (PMC11245831; doi:10.1186/s12944-024-02203-z)
Supplement: Supplementary file 2 — Supplementary Material 2 [file 12944_2024_2203_MOESM2_ESM.pdf]

Petersburg Sleep Quality Index Questionnaire

The following questions are about your usual sleeping habits during the past month. Please read the following questions carefully and choose the right option that suits your situation.

1. During the past month, what time have you usually gone to bed at night?

Bed time ____________

2. During the past month, how long (in minutes) has it usually taken you to fall asleep each night?

Number of minutes ____________

3. During the past month, what time have you usually gotten up in the morning?

Getting up time ____________

4. During the past month, how many hours of actual sleep did you get at night? (This may be different than the number of hours you spent in bed.)

Hours of sleep per night____________

5. During the past month, how often have you had trouble sleeping because you:

a. Cannot get to sleep within 30 minutes

Not during the past month ☐ Less than once a week ☐ Once or twice a week ☐

Three or more times a week ☐

b. Wake up in the middle of the night or early morning

Not during the past month ☐ Less than once a week ☐ Once or twice a week ☐

Three or more times a week ☐

c. Have to get up to use the bathroom

Not during the past month ☐ Less than once a week ☐ Once or twice a week ☐

Three or more times a week ☐

d. Cannot breathe comfortably

Not during the past month ☐ Less than once a week ☐ Once or twice a week ☐

Three or more times a week ☐

e. Cough or snore loudly

Not during the past month ☐ Less than once a week ☐ Once or twice a week ☐

Three or more times a week ☐

f. Feel too cold

Not during the past month ☐ Less than once a week ☐ Once or twice a week ☐

Three or more times a week ☐

g. Feel too hot

Not during the past month ☐ Less than once a week ☐ Once or twice a week ☐

Three or more times a week ☐

h. Had bad dreams

Not during the past month ☐ Less than once a week ☐ Once or twice a week ☐

Three or more times a week ☐

i. Have pain

Not during the past month ☐ Less than once a week ☐ Once or twice a week ☐

Three or more times a week ☐

j. How often during the past month have you had trouble sleeping because of this, other reason(s), please describe?

Not during the past month ☐ Less than once a week ☐ Once or twice a week ☐

Three or more times a week ☐

6. During the past month, how often have you taken medicine to help you sleep (prescribed or "over the counter")?

Not during the past month ☐ Less than once a week ☐ Once or twice a week ☐

Three or more times a week ☐

7. During the past month, how often have you had trouble staying awake while driving, eating meals, or engaging in social activity?

Not during the past month ☐ Less than once a week ☐ Once or twice a week ☐

Three or more times a week ☐

8. During the past month, how much of a problem has it been for you to keep up enough enthusiasm to get things done?

Not during the past month ☐ Less than once a week ☐ Once or twice a week ☐

Three or more times a week ☐

9. During the past month, how would you rate your sleep quality overall?

Very good ☐ Fairly good ☐ Fairly bad ☐ Very bad ☐
