# Supplementary material for: Effects of short-chain fatty acid-butyrate supplementation on expression of circadian-clock genes, sleep quality, and inflammation in patients with active ulcerative colitis: a double-blind randomized controlled trial
Source: Lipids Health Dis. 2024 Jul 13;23:216. doi: 10.1186/s12944-024-02203-z (PMC11245831; doi:10.1186/s12944-024-02203-z)
Supplement: Supplementary file 3 — Supplementary Material 3 [file 12944_2024_2203_MOESM3_ESM.pdf]

Revised-Manuscript.docx

WORD COUNT 9006

TIME SUBMITTED 10-MAR-2024 08:44PM

PAPER ID 107460739

The Effects of Short-Chain Fatty Acid-Butyrate Supplementation on the Expression of Circadian-Clock Genes and Sleep Quality in Patients with Active Ulcerative Colitis: A Double-Blind Randomized Controlled Trial

Donya Firoozi [1], Seyed Jalil Masoumi [2, 3, *], Seyed Mohammad-Kazem Hosseini Asl [4], Aurélie Labbe [5], Iman Razeghian-Jahromi [6], Mohammad Fararouei [7], Kamran Bagheri Lankarani [8], Mahintaj Dara [9]

[1] Student Research Committee, School of Nutrition and Food Sciences, Shiraz University of Medical Sciences, Shiraz, Iran

[2] Nutrition Research Center, School of Nutrition and Food Sciences, Shiraz University of Medical Science, Shiraz, Iran

[3] Gastroenterohepatology Research Center, Shiraz University of Medical Sciences, Shiraz, Iran

[4] Department of Internal Medicine, Gastroenterology Ward, School of Medicine, Shiraz University of Medical Sciences, Shiraz, Iran

[5] Departement of Decision Sciences, HEC, Université de Montréal, QC, Montreal, Canada

[6] Cardiovascular Research Center, Shiraz University of Medical Sciences, Shiraz, Iran

[7] Department of Epidemiology, School of Public Health, Shiraz University of Medical Sciences, Shiraz, Iran

[8] Health Policy Research Center, Institute of Health, Shiraz University of Medical Sciences, Shiraz, Iran

[9] Stem Cells Technology Research Center, Shiraz University of Medical Sciences, Shiraz, Iran

*** Correspondence:**

Corresponding Authors:

Seyed Jalil Masoumi

Email: masoumi7415@gmail.com, sjm@sums.ac.ir

Abstract

Background: The regulation of the circadian clock genes, which coordinate the activity of the immune system, is disturbed in inflammatory bowel disease (IBD). Emerging evidence suggests that butyrate, a short-chain fatty acid produced by the gut microbiota is involved in the regulation of inflammatory responses as well as circadian-clock genes. This study was conducted to investigate the effects of sodium-butyrate supplementation on the expression of circadian-clock genes, inflammation, sleep and life quality in active ulcerative colitis (UC) patients. **Methods:** In the current randomized placebo-controlled trial, 36 active UC patients were randomly divided to receive sodium-butyrate (600 mg/kg) or placebo for 12-weeks. We assessed the expression of circadian clock genes (CRY1, CRY2, PER1, PER2, BMAl1 and CLOCK) by real time polymerase chain reaction (qPCR) in whole blood. Gene expression changes were presented as fold changes in expression (2^-ΔΔCT) relative to the baseline. The faecal calprotectin and serum level of high-sensitivity C-reactive protein (hs-CRP) were assessed by enzyme-linked immunosorbent assay method (ELIZA). Moreover, the sleep quality and IBD quality of life (QoL) were assessed by Pittsburgh sleep quality index (PSQI) and inflammatory bowel disease questionnaire-9 (IBDQ-9) respectively before and after the intervention. **Results:** The results showed that sodium-butyrate supplementation in comparison with placebo significantly decreased the level of calprotectin (-133.82 ± 155.62 vs. 51.58 ± 95.57, p-value <0.001) and hs-CRP (-0.36 (-1.57, -0.05) vs. 0.48 (-0.09-4.77), p-value <0.001) and upregulated the fold change expression of CRY1 (2.22 ± 1.59 vs. 0.63 ± 0.49, p-value < 0.001), CRY2 (2.15 ± 1.26 vs. 0.93 ± 0.80, p-value= 0.001), PER1 (1.86 ± 1.77 vs. 0.65 ± 0.48, p-value= 0.005), BMAL1 (1.85 ± 0.97 vs. 0.86 ± 0.63, p-value= 0.003). Also, sodium-butyrate caused an improvement in the sleep quality (PSQI score: -2.94 ± 3.50 vs. 1.16 ± 3.61, p-value <0.001) and QoL (IBDQ-9: 17.00 ±

11.36 vs. -3.50 ± 6.87, p-value <0.001). **Conclusion:** Butyrate may be an effective adjunct treatment for active UC patients by reducing biomarkers of inflammation, upregulation of circadian-clock genes and improving sleep quality and QoL.

Keywords: Short-chain fatty acids, Butyrate, Circadian-clock genes, Sleep, Inflammation, Ulcerative colitis, IBD, Post-biotic

Background

Inflammatory bowel disease (IBD) is a chronic inflammatory disruption condition characterized by alternating relapse and remission patterns of inflammation of the intestinal mucosa (1). IBD can manifest clinically as Crohn's disease (CD) or ulcerative colitis (UC), which CD affecting the entire gastrointestinal (GI) tract and UC affecting the mucosal tissue of the colon (2). UC and CD are characterized by symptoms, such as acute, and chronic inflammation, diarrhea, rectal bleeding, and abdominal pain (2). Considering its unclear etiology and absence of definite treatment approaches, IBD stands as a prominent subject of study within the realm of gastrointestinal disorders (2). The etiology of IBD may involve a combination of factors, including environmental, nutrition, microbiome, infectious agents, genetic predisposition, and immune response (3). The relevance of chrono-disturbance, also known as circadian rhythm, in comprehending the pathophysiology and etiology of IBD has attracted increasing attention in recent years (4). This is because circadian rhythms play a crucial role to regulate the immune system activity (5), intestinal permeability (6), cytokines production (7), translocation of bacterial endotoxins, and products (8), and gut microbiota composition (9). This rhythm is regulated by the central clock in the hypothalamic suprachiasmatic nucleus (SCN) and peripheral clocks found in most body cells (10). These clocks are controlled at the cellular level by

transcriptional translational autoregulatory feedback loops (10, 11). The core clock genes involved in this process include circadian locomotor output cycles kaput gene (CLOCK) and brain and muscle aryl hydrocarbon receptor nuclear translocator-like1 (BMAL1), which encode transcriptional activators, as well as period 1 (PER1), period 2 (PER2), cryptochrome 1 (CRY1) and cryptochrome 2 (CRY2) which encode transcriptional repressors (10, 11). Intestinal biopsies and peripheral blood mononuclear cells (PBMCs) from IBD patients indicate reduced expression of almost all circadian rhythm genes, including BMAL1, CLOCK, CRY1, CRY2, PER1, and PER2 which this decline was more pronounced in UC than in CD (12-15). These genes show the bidirectional interactions with inflammation (16), and gut microbiota dysbiosis (17), both of which play crucial roles in the development, and progression of IBD. Pro-inflammatory cytokines such as tumour necrosis factor (TNF)-α and interleukin-6 (IL-6), according to studies, decreased the production of PER2, PER1, and CRY2 (16). In contrast, anti-TNF treatment increased the level of PER2 and CRY2 in the immortalized monocyte-like cells (16). Indeed, the levels of these genes in PBMCs show a negative correlation with the levels of inflammatory markers, such as C-reactive protein (CRP), erythrocyte sedimentation rate (ESR), and faecal calprotectin in IBD patients, which indicates a strong and complex relationship among inflammation, circadian-clock genes, and disease activity in these patients (3, 15). Moreover, disruptions of circadian-clock genes can affect the gut microbiota composition which potentially leads to intestinal dysbiosis (17, 18). Research indicates that the expression of central and peripheral circadian-clock genes was shown to be decreased in the brain, liver, and intestinal epithelium of germ-free mice in comparison to normal mice (17). These findings highlight the intricate relationship between the gut and the brain, a connection commonly referred to as the gut-brain axis. Moreover, the dysbiosis seen in UC disease is characterized by diminished levels

of bacteria responsible for butyrate production, such as Faecalibacterium prausnitzii and Roseburia (19) so that the level of butyrate in the stool, and colon of active UC patients is significantly lower than healthy individuals (20, 21).

Butyrate is a short-chain fatty acid (SCFA) which is produced by intestinal butyrate-producing bacteria through fermentation of nonstarch-polysaccharides. Butyrate, as the primary energy source for colonocytes, plays a role in water and electrolyte absorption, contributing to improved faecal consistency and management of diarrhea (22). Furthermore, it is involved in multiple regulatory functions on the intestinal surface and affects the differentiation of intestinal epithelial cells (23), regulates tight junction protein expression (23), enhances the intestinal functional barrier, and increases the expression of antimicrobial mucus, and peptides in intestinal epithelial cells (23). Butyrate has the potential to regulate gut microbiota dysbiosis (24) and inhibit inflammatory responses by decreasing the expression of proinflammatory cytokines via the inhibition of nuclear factor kappa B (NF-κB) transcription factor activation in immune cells (25), as well as increasing the anti-inflammatory cytokine-like IL-18 (26). Furthermore, it can induce epigenetic modifications that control gene expression as well as circadian clock genes by inhibiting histone deacetylases (HDACs) in cells (27).

Recently, increasing evidence has emerged supporting the use of SCFAs, particularly butyrate, as a post-biotic therapeutic approach in managing IBD. However, despite butyrate's significant role in the epigenetic regulation of circadian genes, and its potential to improve the dysbiosis and inflammation, there was no study investigating the effects of butyrate therapy on circadian gene expression in active UC patients. Therefore, this study aims to assess the effect of butyrate supplementation on the upregulation of circadian gene expression and the reduction of

inflammation, as well as evaluate its effect on sleep and life quality in patients with mild to moderately active UC.

Materials and methods

Study design

The study was carried out as a parallel design, placebo-controlled double-blind Randomized Controlled Trial (RCT). Ethical approval for the study was obtained from the Research Ethics Committee of Shiraz University of Medical Sciences, with reference number IR.SUMS.SCHEANUT.REC.1400.037 before enrollment of the first participant in October 2021. Furthermore, the study was registered on IRCT.ir under the registration number IRCT20211214053401N1 on January 1st, 2022 prospectively.

Participants

From January 2022 to February 2023, participants for the study were recruited from UC patients who were referred to the IBD clinic of Shahid Faghihi Hospital, Shiraz University of Medical Sciences, Shiraz, Iran. The recruitment was carried out by gastroenterologists who were members of the clinic. Patients with mild to moderate active UC at the clinic visit, whose disease was confirmed through endoscopic and histological examinations at least three months before the start of study, were consider as eligible. Additionally, participants were required to have a body mass index (BMI) between 18.5 and 30 kg/m2 and to be between 18 and 60 years old.

Exclusion criteria were the use of anti-inflammatory drugs (such as immune-modulators (including 6-mercaptopurine, Azathioprine, Methotrexate and Cyclosporine A), corticosteroids and anti-TNF-α medications (including Infliximab, Adalimumab and Certolizumab pegol)) at

baseline or during the study; the use of antibiotics drugs during or two months before the trial; the use of pro-/pre-/synbiotics, SCFAs, multivitamin-mineral, antioxidant or omega 3 supplements within the last three-months; significant change in diet and lifestyle during the study; change in type or dose of medication during the study; infection with COVID-19 virus during the study or within the last two months prior to recruitment; the use of tobacco and alcohol at the time of enrollment; any addiction to opiates; to have another gastrointestinal, renal, hepatic, cardiovascular, autoimmune, respiratory diseases or diabetes mellitus; history of colostomy and cancer; hospitalization for any reason during the study; pregnancy and lactation and reluctant to participation. Prior to enrollment in the study, eligible participants were required to provide written informed consent.

Sample size determination

According to previous study (28), the sample size was estimated based on mean difference of 178 μg/g in calprotectin levels between the intervention and control groups, considering standard deviations (SD) of 98.46 and 185.43 in the intervention and control groups, respectively. Accounting for a two-sided type I error rate of 0.05 and a type II error rate of 0.10 (power of 90%), the minimum required sample size per group was estimated to be 15 participants in each group which considering a predicted attrition rate of 20%, the sample size was increased to 18 per group. The following formula was used to calculate the required sample size:

$$n = \frac{\left(Z_1 - \frac{\alpha}{2} + Z_1 - \beta\right)^2 \left(S_1^2 + S_2^2\right)}{\left(\mu_1 - \mu_2\right)^2}.$$

Randomization, allocation concealment and blinding

Eligible patients were randomly allocated to either the intervention or placebo groups in a 1:1 ratio. This allocation was performed using block randomization with a block size of 4, using a random number generator available at https://www.sealedenvelope.com/simplerandomiser. The randomization process was conducted by a statistician who had no other involvement in the study. Allocation concealment was maintained by placing the randomization numbers inside sequentially numbered sealed opaque envelopes. These envelopes were opened in consecutive order when patients were admitted to the study. As a result, researchers, participants, and laboratory staff remained completely unaware of the assigned type of supplements in each group until the database was unlocked.

Interventions and Study procedure

The intervention group was administered a 600 mg/d sodium-butyrate capsule throughout the 12-week study period. The sodium-butyrate supplement was provided by Body Bio Company (Body Bio, USA), containing ingredients such as butyric acid, sodium hydroxide, medium-chain triglycerides (MCT), hydroxypropyl methylcellulose, and purified water. The dosage of sodium-butyrate was selected based on the manufacturer's recommended guidelines, which has proven to be safe and free of any side effects in previous trials (29, 30). The control group received a 600 mg/d of rice starch capsules as a placebo for 12 weeks. The placebo capsules were indistinguishable from the active capsules in terms of their shape, size, and color. Participants were instructed to take one capsule daily with their main meal (lunch) in addition to their regular prescribed medications. Participants were also instructed to maintain their regular dietary habits. In addition, IBD patients were asked to follow the dietary recommendations throughout the study

period. Supplements were administered twice at baseline and during the sixth week. To ensure patient compliance and identify any adverse effects, patients were regularly monitored through weekly phone calls. Follow-up visits were scheduled during the sixth week that patients were specifically asked about the positive or negative effects of the supplement, the presence of cramps, and details regarding the frequency, form, and any blood in their stools. Moreover, adherence to the supplement was evaluated by counting the remaining supplements returned by the participants. Participants were deemed compliant if they consumed at least 85% of the provided supplements.

Study outcomes

The primary outcomes variables of this study are the mean reduction in calprotectin levels and the mean increase in the expression of circadian clock genes (CRY1, CRY2, PER1, PER2, CLOCK, and BMAL1) over a 12-weeks period following sodium-butyrate supplementation, compared to a placebo. Secondary outcomes include improvements in sleep and life quality and the mean decrease in hs-CRP levels within the group receiving sodium-butyrate, compared to the placebo group, after the 12-weeks period.

Demographic, dietary, and anthropometric assessment

Participants' general demographics, medical history, disease extent and duration were collected by a questionnaire. To assess dietary habits, participants were asked to complete three 24-hour analogue food records, documenting their intake for two weekdays and one weekend day, both at the beginning and at the end of the study period. To determine daily calorie and nutrient intake, Nutritionist IV software (First Databank, San Bruno, CA, USA) modified for Iranian food, was

used. Body weight and height were measured by using a Seca scale (Seca, Germany) and wall-mountable height rod respectively at the beginning and end of the study. BMI was then computed by dividing weight (in kilograms) by the square of height (in meters).

Blood and stool sampling and biochemical assessment

Blood samples (15 ml) and stool sample (10 gr) were collected at the beginning and end of the study, following a 10-12 hour fast between 7:30-8:30 am. Out of 15 ml of whole blood that was obtained from the participants, 10 ml was allocated to biochemical analyses and the remaining of 5 ml was subjected to total RNA extraction for subsequent gene expression analysis which kept refrigerated at -80 ˚C. To isolate the serum, the blood samples were centrifuged at room temperature at 3000 rpm for 10 minutes. The separated serum was then stored at -80 °C until the biochemical tests could be conducted. For the serum glutamic-oxaloacetic transaminase (SGOT) and serum glutamate pyruvate transaminase (SGPT) concentrations, routine enzymatic assays with commercial kits (Pars Azmoon, Tehran, Iran) were used with a chemistry autoanalyzer (BT1500, Biotecnica, Italy). Complete blood count (CBC) analysis was performed using the automated analyzer (XS-500i, Sysmex, Japan). The level of hs-CRP was measured using enzyme-linked immunosorbent assay (ELISA) methods by Zellbio kit, Germany. To determine the fecal concentration of calprotectin, weighing method was used to extract calprotectin from stool sample. Based on this method, 40 mg of stool sample was mixed with 1960 microliters of extraction buffer (1:50 ratio) and homogenized for 25 minutes. Subsequently, one milliliter of the homogenate was transferred to a tube and centrifuged for 20 minutes. The resulting supernatant was collected and frozen at -20°C. The analysis of calprotectin was carried out using an ELISA kit (Pishtaz Co., Iran).

RNA extraction and real-time PCR analysis

For gene expression analysis, 5 ml of whole blood which was obtained from the participants and kept refrigerated at -80 ˚C. In the molecular lab, the samples were thawed. RBC was removed using lysis buffer, and white blood cells (buffy coat), as starting material, were subjected to total RNA extraction (Rnx Plus Solution, CinnaGen Co., Iran). The quantity and quality of the extracted RNA were assessed using a nanodrop instrument (Thermo Scientific, NanoDrop 2000c, US), ensuring that all RNA samples had a 260:280 absorbance ratio. To synthesize cDNA, the AddScript cDNA Synthesis Kit (Add bio, South Korea) was used following the manufacturer's instructions. The resulting cDNA was stored at -80°C until further analysis. Real-time quantitative polymerase chain reaction (qPCR) was performed to determine the expression levels of genes in the extracted cDNA samples. For qPCR, specific primers were utilized, and the RealQ Plus 2x Master Mix Green (Ampliqon, Denmark) was employed, with the reaction carried out using the StepOne thermocycler (Applied Biosystems, US). The primers were designed using AlleIID software, V 7.50 (Primier Biosoft International, US), and the specificity of the primers was confirmed using Primer-BLAST (https://www.ncbi.nlm.nih.gov/tools/primer-blast/). The housekeeping gene, Β-actin, was used as a reference (Table 1). Following each qPCR run, melt curve analysis was performed on all samples to ensure the specificity of the reaction. Gene expression changes were presented as fold changes in expression ($2^{\wedge-\Delta\Delta CT}$) relative to the baseline, which was normalized to β-actin as the reference gene.

Table 1

Sequence of genes primers for Real-timePCR.

Gene	Primer sequence (5′ to 3′)
CRY1	Forward GGAAGAAGGAATGAAGGTAT<br>Reverse TAGGACAGGCAAATAACG
CRY2	Forward TCCCAAGGCTGTTCAAGG<br>Reverse ATTCTCCGTCACTACTTCCA
PER1	Forward GTGCGGAGGCTGCTGAG<br>Reverse GTTGGTGTTGAGGAAGG
PER2	Forward ATTGTGAAGAATGCCGATA<br>Reverse GAGGTGAAACTGTGGAAC
BMAL1	Forward CCAATCCATACACAGAAGCAA<br>Reverse CCTCGGTCACATCCTACG
CLOCK	Forward ACGAGAACTTGGCATTG<br>Reverse GTTGGTGTTGAGGAAGG
B-actin (HKG*)	Forward GTGGGCATGGGTCAGAAG<br>Reverse GGGTACTTCAGGGTGAGGA

*HKG: Housekeeping gene

Disease severity, sleep and life quality assessment

The severity of UC was evaluated using the 9-point partial Mayo score which individuals with scores ranging between 2 and 7 were selected for this study as a mild to moderate active UC (31). For assessing sleep quality over the last month, the Persian version Pittsburgh Sleep Quality Index (PSQI) was used at the baseline and end of study by face-to-face interview (32). This questionnaire consists of 19 items categorized into seven components, which include sleep quality, sleep latency, sleep duration, habitual sleep efficiency, sleep disturbance, use of sleeping medication, and daytime dysfunction. Each component is weighted equally on a scale of 0-3. The scores for all components are summed to obtain the global score, which ranges from 0 to 21. Higher scores indicate poorer sleep quality, and a global score of 6 or higher indicates bad sleep quality. Patients' quality of life (QoL) was evaluated using the Inflammatory Bowel Disease Questionnaire-9 (IBDQ-9) at the baseline and end of study by face-to-face interview (33). This questionnaire consists of 9 items that assess 4 dimensions related to bowel and systemic

symptoms, emotional status, and social functioning. Each item is scored from 1 to 7, with a total score ranging from 9 to 63. Higher scores indicate a better quality of life.

Statistical analysis

Statistical analyses were conducted using the R software version 4.2.2 (R Development core team, R Foundation for Statistical Computing, Vienna, Austria). All data analysis were undertaken using the intention-to-treat (ITT) principle based on the "the last value carried forward" protocol. Before conducting bivariate analysis, the distribution of quantitative variables is assessed using the Shapiro-Wilk test, along with normality curves. Mean (SD) and median with first and third quartiles (Q1, Q3) are used to describe normally and non-normally distributed variables respectively. Categorical variables are described using frequencies and percentages. Baseline patient characteristics and dietary intake with normal and non-normal distribution are compared between the two groups using an independent sample t-test or Mann-Whitney U test respectively. For categorical variables, Chi-square or Fisher's exact tests were applied, as appropriate. Within-group comparisons are conducted using paired sample t-test and Wilcoxon signed ranked test in the case of normally and non-normally distributed variables respectively. For the evaluation of between-group comparisons, an independent sample t-test or Mann-Whitney U test for normally and non-normally distributed variables, respectively was utilized. Also, multiple linear regression was employed to address potential confounders by adjusting for baseline outcome values, age, sex and baseline PSQI (for circadian genes). Based on previous studies, age and sex are potential confounding factors in patients with IBD, as they can significantly influence circadian rhythms and inflammatory pathways, thus may impacting treatment response (34-37). Given that multiple outcomes were tested simultaneously (7 primary

outcomes and 7 secondary outcomes), p-values were corrected for multiple testing using a Bonferroni correction applied separately to primary and secondary outcomes. Consequently, the significance p-value thresholds were set at 0.007 for primary and secondary outcomes, respectively, based on a type I error rate of 5%. For another data analysis p-value ≤ 0.05 was used to determine statistical significance. Prism software version 8.0 (GraphPad, CA, USA) was used for drawing figures.

Results

Baseline characteristics of participants and dietary intake

According to the CONSORT flowchart of study (Fig. 1), a total of 201 referred patients were assessed, resulting in the randomization of 36 eligible subjects (16 women and 20 men) with mild to moderate active UC into either the intervention or placebo groups. Two individuals dropped out from the intervention group due to low compliance rate (n=1) and COVID-19 (n=1) and four individuals dropped out the placebo group due to gastrointestinal complain (n=2) and worsen UC state (n=2). In the end, a total of thirty-six participants were included in the final analysis (Fig.1). Table 2 presents the baseline demographic and laboratory characteristics of the patients in the butyrate and placebo groups. The mean age of sodium-butyrate and placebo groups were 41.16 (10.95) and 38.16 (12.38) years consequently. Participants baseline demographic and laboratory characteristics in both groups were similar, and it is important to note that all patients received consistent anti-inflammatory treatment (Table 2).

Dietary habits were assessed to ensure participants' diets remained consistent before and after the intervention. Table 3 displayed the baseline and 12-week measurements of energy,

macronutrients, and dietary fiber intake. A comparison between the two groups indicated that there were no significant differences in these parameters at either the baseline or the 12-week intervention ($p > 0.05$).

Fig 1. CONSORT flowchart of design and protocol of study.

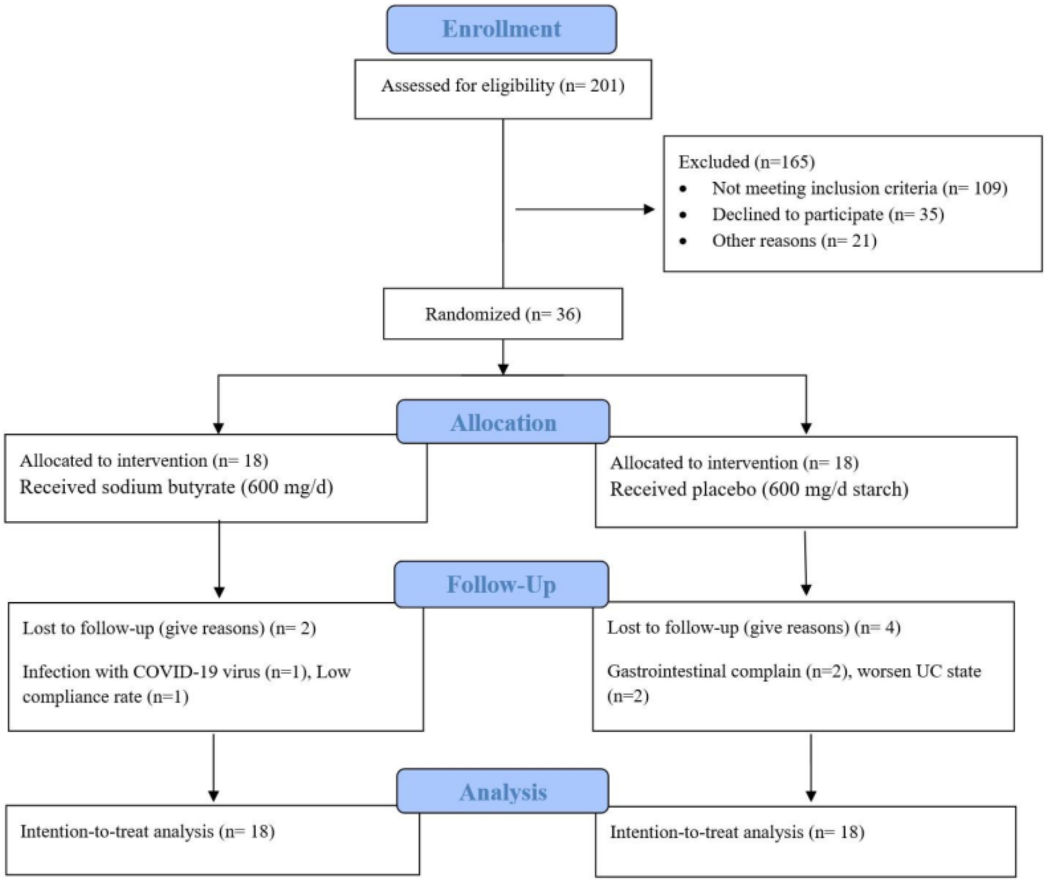

Table 2

Baseline characteristics of participants in the sodium-butyrate-treated and placebo groups.

variables	Groups		p-value
	Butyrate (n=18)	Placebo (n=18)	
Age (years)	41.16 (10.95)	38.16 (12.38)	0.44 #
Sex n (%)			0.73
Female	7 (38.90)	9 (50.00)	
Male	11 (61.10)	9 (50.00)	
BMI (kg/m^2)	23.01 (20.44-27.66)	22.24 (20.06-27.42)	0.88 #
Weight (kg)	63.50 (60-75.25)	62.50 (58.00-80.00)	0.74 #
Disease duration (years)	5.6 (4.1)	4.02 (3.23)	0.24 #
Married state n (%)			0.17 ¥
Married	13 (72.20)	8 (44.4)	
Single	5 (27.80)	10 (55.55)	
Education n (%)			0.74 ¥
Diploma or lower	12 (66.70)	10 (55.60)	
Academic	4 (22.20)	6 (33.30)	
Post-graduate	2 (11.10)	2 (11.10)	
Current treatment n (%)			0.73 ¥
Aminosalicylates	9 (50.00)	7 (38.90)	
Aminosalicylate and asacole suppositories or enema	9 (50.00)	11 (61.10)	
SGOT (U/L)	18.70 (14.75-22.25)	19.00 (16.25-23.12)	0.58 #
SGPT (U/L)	17.00 (9.87-25.62)	19.00 (13.75-25.25)	0.79 #
Hemoglobin (g/dL)	13.20 (12.47-14.07)	13.10 (12.15-14.88)	0.74 #
WBC count (10^9/L)	8.15 (5.87-10.22)	7.90 (5.95-9.90)	0.65 #
Platelet count (10^9/L)	340.83 (78.99)	314.72 (90.96)	0.36 #
Partial Mayo score	3.50 (2.00-5.00)	3.50 (3.00-4.00)	0.76 #

The data were reported as mean (SD) and median (Q1- Q3) for normally and non-normally distributed quantitative variables, respectively and frequency (%) for qualitative variables.

# p-values obtained from independent sample t-test or Mann-Whitney U test for comparisons between group analysis for normally and non-normally distributed quantitative variables, respectively.

¥ p-values obtained from Chi-square or Fisher's exact test for comparisons between group analysis of qualitative variables.

$P \leq 0.05$ was considered as statistically significant.

BMI: body mass index; SGOT: serum glutamic-oxaloacetic transaminase; SGPT: serum glutamate pyruvate transaminase; WBC: white blood cell.

Table 3

Dietary intake of participants at baseline and after 12 weeks of intervention.

Variable		Butyrate (n=18)	Placebo (n=18)	p-value [¥]
Energy (kcal/d)	Pre	2033.23 (1791.59-2351.43)	2383.28 (681.78)	0.90
	Post	1998.27 (1714.54- 2463.61)	2345.85 (676.44)	
	Change	-55.57 (482.07)	-37.42 (447.41)	
	p-value [≠]	0.83	0.73	
Carbohydrate (g/d)	Pre	290.28 (199.06-346.27)	277.18 (256.03- 340.61)	0.73
	Post	268.44 (221.10- 359.85)	258.28 (193.98- 388.96)	
	Change	-16.10 (73.37)	-25.02 (79.68)	
	p-value [≠]	0.28	0.11	
**Protein (g/d)**	Pre	67.51 (37.43- 108.38)	81.16 (33.40)	0.76
	Post	74.98 (68.63- 118.94)	92.16 (34.94)	
	Change	7.59 (35.80)	11.00 (29.85)	
	p-value [≠]	0.33	0.14	
Fat (g/d)	Pre	69.81 (43.93- 82.21)	76.56 (69.01- 108.09)	0.72
	Post	61.35 (43.20- 77.61)	80.13 (61.04- 127.74)	
	Change	-3.03 (35.21)	1.00 (31.98)	
	p-value [≠]	0.22	0.58	
Total fiber (g/d)	Pre	13.81 (9.41- 19.41)	19.51 (8.11)	0.65
	Post	11.52 (8.26- 22.67)	17.62 (8.21)	
	Change	-0.14 (7.81)	-1.5 (10.03)	
	p-value [≠]	0.94	0.54	

The data were reported as mean (SD) and median (Q1, Q3) for normally and non-normally distributed variables, respectively.

Change calculated as: (post-intervention – baseline) in each study group.

[≠] p-values obtained from paired samples t-test or Wilcoxon signed ranked test for comparison within group to test before/after treatment.

[¥] p-values were obtained by comparing the change values using independent sample t-test or Mann-Whitney U test for comparisons between group analysis for normally and non-normally distributed quantitative variables, respectively.

[*] p-value ≤ 0.05 was used to determine statistical significance.

p-value for the between group analysis is obtained by comparing the change values.

Butyrate decreased the level of fecal calprotectin in patients with UC

The between-group analysis demonstrated that the level of fecal calprotectin significantly decreased (p-value <0.001) in the sodium-butyrate group compared to the placebo group based on independent sample t-test (Table 4). However, based on multiple regression which adjusted for baseline level of calprotectin, age and sex no differences were observed in the results (β = -173.88, p-value <0.001). Moreover, according to within group analysis, the level of fecal calprotectin significantly decreases in the sodium-butyrate group during treatment (p-value= 0.002). However, in the placebo group, no significant changes were observed (Table 4).

Butyrate modulated circadian-clock genes expression in patients with active UC

We found a significant difference between the groups in terms of the impact of sodium-butyrate on changes in gene expression from baseline to post-intervention, as indicated by independent sample t-tests or Mann-Whitney U tests. In the sodium-butyrate group compared to the placebo group, the change in expression level of CRY1 was more than three-fold higher (CRY1fold change: 2.22 ±1.59 vs. 0.63 ±0.49, p-value < 0.001). Similarly, the changes in expression levels of CRY2, PER1, and BMAL1 were more than two-fold higher (CRY2 fold change: 2.15 ±1.26 vs. 0.93 ±0.80, p-value= 0.001), (PER1 fold change: 1.86 ±1.77 vs. 0.65 ±0.48, p-value= 0.005), and (BMAL1 fold change: 1.85 ±0.97 vs. 0.86 ±0.63, p-value= 0.001), respectively. PER2 and CLOCK exhibited an increasing pattern, although the observed trends were not statistically significant (Fig.2). However, after adjusting for baseline PSQI, age, and sex using multiple linear regression, no significant differences were observed in the results ((CRY1fold change: β =1.36, p-value < 0.001), (CRY2 fold change: β =1.17, p-value= 0.001), (PER1 fold change: β =1.12, p-value= 0.005), and (BMAL1 fold change: β =0.91, p-value=

0.003)). Also, within-group analysis revealed that in the sodium-butyrate group, significant increases were observed in the levels of CRY1 (p-value= 0.003), CRY2 (p-value= 0.001), BMAL1 (p-value= 0.002), and CLOCK (p-value= 0.006) during the 12 weeks intervention (Fig.2). In the placebo group, no significant changes were observed in the expression levels of most genes, except for CRY1 (p-value= 0.006) and PER1 (p-value= 0.007), which show a significant decrease during the treatment (Fig.2).

Table 4.

Outcome variables at baseline and after 12 weeks of intervention.

Variables		Butyrate (n=18)	Placebo (n=18)	p-value ¥	Adjusted p-value §
CALPR	Pre	326.91 (291.73- 424.83)	313.99 (214.79-405.85)	<0.001*	<0.001*
	Post	224.39 (45.93-307.16)	387.70 (322.20-426.71)		
	Change	-133.82 (155.62)	51.58 (95.57)		
	p-value ≠	0.002 *	0.02		
hs-CRP	Pre	2.23 (0.94- 3.66)	3.18 (2.15-5.64)	0.009	<0.001*
	Post	1.47 (0.42-2.89)	4.58 (1.96-10.57)		
	Change	-0.36 (-1.57, -0.05)	0.48 (-0.09- 4.77)		
	p-value ≠	0.003 *	0.03		
PSQI score	Pre	9.61(5.00)	10.44 (4.46)	0.001*	<0.001*
	Post	6.66 (3.39)	11.61 (4.80)		
	Change	-2.94 (3.50)	1.16 (3.61)		
	p-value ≠	0.002 *	0.18		
IBDQL scores					
Systemic	Pre	9.77 (3.93)	9.27 (4.42)	<0.001*	<0.001*
	Post	15.94 (3.42)	8.77 (4.20)		
	Change	6.16 (4.20)	-0.50(2.30)		
	p-value ≠	<0.001 *	0.30		
Intestinal	Pre	13.55 (5.07)	14.33 (4.32)	<0.001*	<0.001*
	Post	22.66 (4.61)	12.11 (4.61)		
	Change	9.11 (6.22)	-2.22 (4.23)		
	p-value ≠	<0.001 *	0.04		
Social	Pre	3.88 (1.56)	3.66 (0.89)	0.04	0.005 *
	Post	4.50 (1.29)	3.61 (0.91)		
	Change	0.61 (0.97)	-0.05 (0.93)		
	p-value ≠	0.01	0.80		
Emotional	Pre	5.11 (1.77)	5.33 (1.97)	0.001*	0.001 *
	Post	6.16 (1.66)	4.77 (2.29)		
	Change	1.05 (1.62)	-0.55 (1.29)		
	p-value ≠	0.01	0.08		
Total	Pre	32.23 (10.30)	32.61 (9.66)	<0.001*	<0.001*
	Post	49.33 (9.17)	29.11 (10.08)		
	Change	17.00 (11.36)	-3.50 (6.87)		
	p-value ≠	<0.001 *	0.04		

The data were reported as mean (SD) and median (Q1, Q3) for normally and non-normally distributed variables, respectively.

Change calculated as: (post-intervention – baseline) in each study group.

≠ p-values were obtained from paired samples t-test or Wilcoxon signed ranked test for comparison within group to test before/after treatment.

¥ p-values were obtained by comparing the change values using independent sample t-tests or Mann-Whitney U tests for comparison between groups analysis.

§ Adjusted p-values were obtained from the multiple linear regression test, which was adjusted for baseline values, age and sex for comparisons between group analysis.

* p-value ≤ 0.007 was used to determine statistical significance using the Bonferroni correction method

CALPR: calprotectin; hs-CRP: High-sensitivity C-reactive protein

Fig 2. The effect of intervention on circadian clock genes expression.

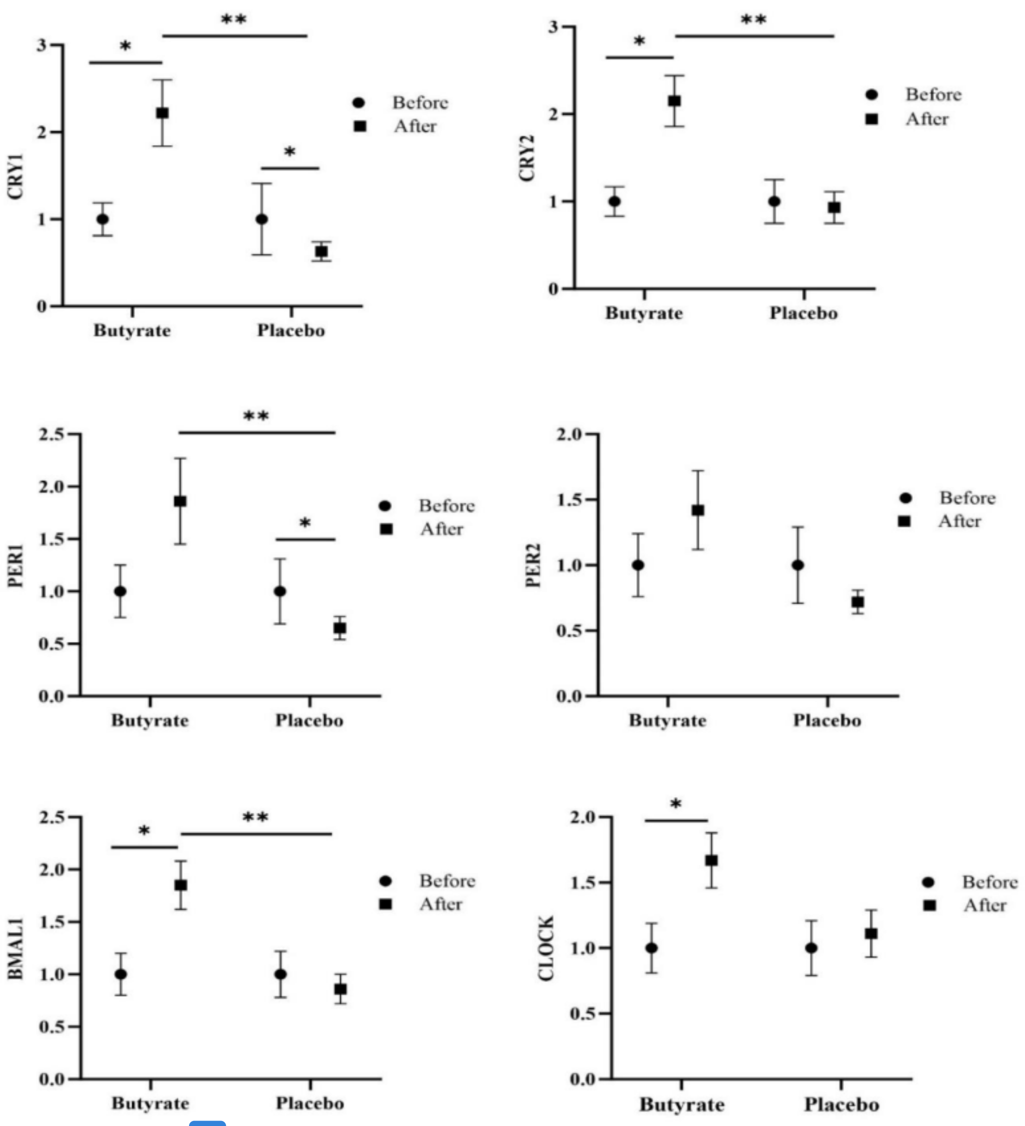

The data were reported as mean (s.e.m) fold change ($2^{\wedge - \Delta\Delta Ct}$) relative to baseline.

p-values were obtained from the multiple linear regression test, which was adjusted [illegible]r baseline PSQI, age and sex for comparisons between group analysis (** p-value ≤ 0.007) and one sample t test for comparisons within group analysis (* p-value ≤ 0.007).

p-value ≤ 0.007 was used to determine statistical significance using the Bonferroni correction method.

Butyrate reduced hs-CRP in patients with active UC

The between-group analysis revealed that the level of hs-CRP did not decrease significantly more in the sodium-butyrate group compared to the placebo group, as indicated by independent sample t-test (p-value=0.009) (Table 4). However, based on multiple regression which adjusted for baseline level of hs-CRP, age and sex the level of hs-CRP decreased significantly in the sodium-butyrate group compared to the placebo group (β = -1.39, p-value <0.001) (Table 4). Moreover, within group analysis showed that the levels of serum hs-CRP significantly (p-value= 0.003) decreased during the intervention in the sodium-butyrate group. However, in the placebo group, no significant changes were observed (Table 4).

Butyrate improved sleep and life quality in patients with UC

Table 4 presents the changes in sleep quality and the overall QoL. The between-group analysis indicated a significant and more substantial decrease (p-value=0.001) in the PSQI score and a significant improvement in the IBDQ parameters' scores, including intestinal (p-value < 0.001), systemic (p-value < 0.001), emotional (p-value =0.001) function, and total (p-value < 0.001) among the sodium-butyrate group compared to the placebo group through the 12-weeks treatment intervention based on independent sample t-test (Table 4). However based on multiple linear regression which adjusted for baseline value, age and sex the results remained significant (PSQI: β=-4.39, p-value < 0.001), (IBDQ intestinal: β=10.81, p-value <0.001), (IBDQ systemic: β=20.58, p-value < 0.001), (IBDQ emotional: β=1.63, p-value= 0.001), (IBDQ social: β=0.83, p-value=0.005) and (IBDQ total: β=1.50, p-value < 0.001) (Table 4). Furthermore, within-group analysis revealed a significant reduction (p-value= 0.002) in the PSQI score and significant improvement in the IBDQ intestinal (p-value < 0.001), systemic (p-value < 0.001)

function, and total (p-value < 0.001) scores in the sodium-butyrate group before and after treatment. However, in the placebo group, no significant changes were observed in these scores before and after the intervention.

Safety

None of the patients who participated in the study reported any significant major or minor adverse effects to the sodium-butyrate intervention.

Discussion

Today, it is understood that circadian disturbance has a role in the onset and severity of several clinical and pathological conditions, including IBD (19), sleep disorders, cancer, depression, metabolic syndrome, and inflammation (20). As a result, research has concentrated on adjunctive and non-invasive chrono-therapeutic ways for treating a variety of diseases. The ability of chrono-nutrition to control the circadian rhythm via dietary patterns and bioactive components offers great promise in this context. Butyrate as a bioactive component, which produce from fiber by gut microbiota, has the ability to alter circadian rhythm, inflammation, and immune system modulation (21). Butyrate can be absorbed in both the small and large intestine through passive diffusion and active transport mechanisms (38). Hence, the administration of oral butyrate supplementation can be considered as a strategy to tackle issues such as insufficient response to increased dietary fiber intake, diminished abundance of butyrate-producing bacteria, and potential obstacles in butyrate transportation and metabolism among individuals with IBD (39). Untreated butyrate is primarily absorbed in the upper gastrointestinal tract. However, in the supplement formula, the butyrate powder is enterically coated to ensure its survival through

exposure to stomach acid. This coating facilitates its passage to the colon, where it can be most effective in supporting gut cells (40).

Considering these facts, we focused for the first time on the effects of butyrate supplementation on the expression of circadian-clock genes in patients with UC. Moreover, our study encompassed an evaluation of the effect of this supplement on calprotectin and hs-CRP levels, as well as on the quality of sleep and life. We identified that sodium-butyrate modulated the expression of circadian-clock genes and decreased inflammation as well as enhanced sleep quality and overall QoL in the patients with UC.

According to our research, sodium-butyrate supplementation for 12 weeks increased the expression of the CRY1, CRY2, PER1, and BMAL1 genes in individuals with mild-to-moderate active UC when compared with placebo. Similar trends were found for PER2 and CLOCK genes but these did not change significantly in response to sodium-butyrate as compared with placebo. No oral sodium butyrate supplementation studies in humans examining the influence on circadian-clock gene expression in UC disease have been found. According to the findings of animal research, giving mice an intraperitoneal injection of butyrate for five days enhanced the PER2/BMAL1 ratio in their livers and mediobasal hypothalamus. Moreover, treatment with butyrate synchronized the rhythm of PER2/BMAL1 expression in hepatocytes (41). Another study revealed that the oral administration of a mixture of SCFAs including butyrate and lactate at various doses (low dose: 200 mM or high dose: 400 mM each), as well as single administrations of these compounds during the midday period for three consecutive days, led to a phase-advancement of peripheral clocks in vivo. All clock genes, including CRY1, PER1, PER2, BMAL1, and Rev-erbα in the kidney, as well as CRY1 and PER2 in the submandibular gland,

exhibited an advanced peak phase in response to treatment (42). Moreover, a study on mice model of Sjögren's syndrome showed that administering intraperitoneal injections of sodium butyrate at a dose of 1 gr/kg, three times weekly regulated the circadian-clock-related genes in Human Submandibular Gland (HSG) cells. The treatment led to an increase in the levels of CRY1, and it induced alterations in the cycling pattern of BMAL1 in HSG cells (43).

The modulatory effect of butyrate on host circadian rhythms can be attributed to several underlying mechanisms. One of the main mechanisms involves the induction of epigenetic regulation on circadian-clock gene rhythms by butyrate through the inhibition of NAD^+-dependent histone deacetylases (HDAC). This inhibition has a notable impact on the modulation of circadian clock gene expression in the intestinal epithelial (44). Another potential mechanism involves SCFA-induced insulin release via the binding to G protein-coupled receptors (GPCRs), which could potentially improve peripheral clock entrainment (45). Insulin induces a phase advance in the rhythm of PER2 gene expression as well as the increase of PER2 gene expression within the mouse liver and embryonic fibroblast cells (46). Another potential mechanism is that the peripheral clocks' phase might be reset as a result of the SCFA-induced change in local pH. The capacity to reset circadian clocks in in vitro tests using an alkali shock (increasing pH by 0.4 units in the medium) has been shown. This action is mediated by the transforming growth factor-β (TGF- β) signaling pathway. This shows that SCFA may be able to reset a pH-mediated clock in the colon or caecum (47).

It has long been accepted clinically that IBD patients often have sleep disorders and have poor sleep quality. This decrease in sleep quality not only makes gastrointestinal symptoms worse but also hastens the illness, causes anxiety and depression, and significantly lowers the patients'

general quality of life. The sleep disorders observed in these patients can be attributed to the dysregulated expression of circadian genes (15). In addition, a fascinating interaction was discovered between sleep quality, inflammation, and the gut microbiota (48) such that sleep deficiency lead to a decrease in the abundance of butyrate-producing bacteria (49). Our research has revealed that the supplementation of sodium butyrate yields to improvement in sleep and life quality by decreasing the PSQI score and increasing the IBDQ-9 scores respectively. Also, we found negative correlation between PSQI and IBDQ-9. This positive outcome may be attributed to its effect on modulating circadian clock genes, along with its capacity to mitigate inflammation and restore balance in the gut microbiota. A prior investigation has also shown the importance of butyrate, a vital metabolite synthesized by intestinal microbiota, in the improvement of sleep quality (50). Moreover, a recent study showed that butyrate effectively alleviates the intestinal mucosal damage caused by insufficient sleep, both in humans and mice. This underscores the potential therapeutic effect of butyrate in mitigating the adverse effects of sleep deprivation on the gastrointestinal system (49). Furthermore, the administration of probiotics, such as VSL3, may directly impact sleep quality and structure by elevating the levels of butyrate (16).

In UC patients, faecal calprotectin and serum hs-CRP are the most used inflammatory markers for assessing the activity of the disease and the effectiveness of treatment. There is a direct correlation among these biomarkers by pro-inflammatory cytokines like IL-6. Calprotectin is a calcium-binding protein that is present in neutrophilic granulocytes. Elevated levels of calprotectin are often a response to neutrophil infiltration into inflamed gut tissue, making it a valuable marker for gauging the extent of intestinal inflammation and prediction of relapses (51, 52).

We found that upon administering oral sodium-butyrate supplementation, significant reductions were observed in faecal calprotectin and serum hs-CRP levels, accompanied by an enhancement in the QoL score. Based on these findings, research conducted on animals has indicated that the administration of butyrate potentially ameliorated inflammation in the model of DSS colitis (53). Furthermore, several clinical studies involving the patients with active UC have demonstrated the beneficial therapeutic effect of butyrate for alleviating gut inflammation when used in conjunction with standard therapy (54, 55). A previous study reported that the administration oral microencapsulated sodium butyrate (1800 mg/day) in UC patients, reduced inflammatory markers including calprotectin, improved QoL and Mayo score by increasing the level of butyrogenic colonic bacteria (56). Furthermore, one study showed that add-on therapy microencapsulated sodium butyrate (500 mg/day) induced remission phase maintenance and decreased the level of faecal calprotectin in UC patients (57). Moreover, the administration of FEEDColon® (a combination of calcium butyrate, probiotics, and fructo-oligosaccharides) with 5-ASA reduced faecal calprotectin and improved QoL and symptoms of patients (58). Another study's findings revealed that administration of oral calcium butyrate plus inulin in conjunction with mesalazine in UC patients led to an elevation in both the butyryl-CoA: acetate CoA-transferase gene levels within the faecal microbiota and the presence of faecal butyrate-producing bacteria. Additionally, levels of serum pro-inflammatory biomarkers decreased, and improvements were noted in terms of rectal bleeding and stool frequency when compared with mesalazine treatment alone (59). Moreover, a recent case study revealed that administration of oral butyrate supplementation to a patient with pharmacologically, and nutritionally resistant UC led to a decrease in faecal calprotectin levels and stool frequency. Furthermore, the patient experienced an enhancement in their QoL, and successfully sustained a state of remission (39).

Butyrate's anti-inflammatory properties can be demonstrated via multiple mechanisms. As an HDAC inhibitor, butyrate is able to counteract the inflammatory response by inhibiting the activation of NF-κB and the production of pro-inflammatory mediators. Additionally, butyrate has the power to activate the nuclear receptor peroxisome proliferator-activated receptor (PPAR) γ, which inhibits NF-κB and has anti-inflammatory actions (60). The anti-inflammatory cytokine IL-18 is produced by butyrate, and intestinal epithelial cells produce less pro-inflammatory cytokines such as TNF-α, IL-1, and IL-8 as a result (60). Finally, butyrate furthers its anti-inflammatory activities by inhibiting the production of chemokines (C-X-C motif) ligand (CXCL)5 and CXCL11 (60).

Strengths and limitations of the study

This research is the first double-blind, placebo-controlled clinical investigation that demonstrates the impact of post-biotic butyrate supplementation on circadian-clock gene expression, to the best of our knowledge. To avoid false positive conclusions regarding the effect of butyrate supplementation, we used a Bonferroni correction for the p-values of the statistical tests to account for the number of tests performed. The study's limited sample size of UC patients, absence of examination of gut microbiota composition and faecal butyrate levels, and inclusion of inflammatory cytokines like IL-6, TNF-α, etc. are only a few of the shortcomings that should be considered. These limitations are due to funding limitations. The limitation of assessing circadian gene expression at a single time point is acknowledged as another weakness in this study. We underscore the importance of future research that incorporates multiple time points (e.g., 7 am, 12 am, 5 pm and 11pm) to achieve a comprehensive understanding of circadian gene dynamics.

Conclusions

In active UC patients, a 12-week supplementation of sodium-butyrate, in conjunction with regular medication, led to a reduction in inflammation as compared with placebo, evidenced by decreased levels of faecal calprotectin and serum hs-CRP in the sodium-butyrate group. Additionally, sodium-butyrate supplementation increased the patients' quality of sleep and overall quality of life. The upregulation of CRY1, CRY2, PER1, and BMAL1 by sodium-butyrate group imply that butyrate supplementation improves the clinical aspects for UC patients through regulation of circadian clock genes. However, this study was the first clinical trial and additional research is essential to corroborate these findings.

Abbreviations

IBD: Inflammatory Bowel Diseases, UC: Ulcerative Colitis, ELIZA: Enzyme-linked immunosorbent assay, CD: Crohn Disease, GI: Gastrointestinal, CLOCK: Circadian locomotor output cycles kaput gene; BMAL1: Brain and muscle aryl hydrocarbon receptor nuclear translocator-like1; PER1/2: Period 1/2, CRY1/2: cryptochrome 1/2; PBMCs: Peripheral blood mononuclear cells; TNF-α: Tumor necrosis factor-α; IL-6: interleukin-6; CRP: C-reactive protein; ESR: Erythrocyte sedimentation rate; SCFAs: short-chain fatty acids; NF-κB: Nuclear factor kappa B; HDACs: histone deacetylases; RCT: Randomized Controlled Trial; BMI: body mass index; PCR: Polymerase chain reaction; QoL: Quality of life; PSQI: Pittsburgh Sleep Quality Index; IBDQ-9: Inflammatory Bowel Disease Questionnaire-9; ITT: intention-to-treat; SD: Standard deviation; GPCRs: G protein-coupled receptors; TGF- β: Transforming growth factor- β; CXCL: C-X-C motif chemokine

Declarations

Ethics approval and consent to participate

Ethical approval for the study was obtained from the Research Ethics Committee of Shiraz University of Medical Sciences, with reference number IR.SUMS.SCHEANUT.REC.1400.037 and it was registered on IRCT.ir under the registration number IRCT20211214053401N1 prospectively. Also, Prior to enrollment in the study, eligible participants were required to provide written informed consent.

Data availability

The data will be available from the corresponding author upon request.

Conflicts of interest

The authors declare that there are no conflicts of interest.

Funding statement

This research was supported by Iran National Science Foundation: INSF grant (Research No. = 4004581).

Authors' Contributions

DF: Designed the research, Methodology, Investigation, Writing – original draft; SJM: Designed the research, Supervision, Writing – review & editing; SMKHA: Designed the research, Data collection; KBL: Designed the research, Data collection; AL: Statistical analysis, Writing –

review & editing; IRJ: Methodology, MD: Methodology; MF: Designed the research, Methodology, Statistical analysis.

Acknowledgements

The authors would like to thank Dr. Hamid Reza Mosalaee poor (Department of Internal Medicine, School of Medicine, Shiraz University of Medical Sciences, Shiraz, Iran) and Mr. Amir Hossein Hadaegh (Department of Medical Genetics, Shiraz University of Medical Sciences, Shiraz, Iran) for their valuable contribution in participant recruitment and genes analysis, respectively.

References

1. Zhang Y-Z, Li Y-Y. Inflammatory bowel disease: pathogenesis. *WJG*. (2014) 20:91-99.

2. Firoozi D, Nekooeian AA, Tanideh N, Mazloom Z, Mokhtari M, Sartang MM. The healing effects of hydroalcoholic extract of Carum copticum L. on experimental colitis in rats. *IJMS*. 2019;44:501-510.

3. Weintraub Y, Cohen S, Anafy A, Chapnik N, Tsameret S, Ben-Tov A, et al. Inverse Relationship Between Clock Gene Expression and Inflammatory Markers in Ulcerative Colitis Patients Undergoing Remission. *Dig. Dis. Sci*. 2023;68:2454–62.

4. Swanson GR, Burgess HJ. Sleep and circadian hygiene and inflammatory bowel disease. *Gastroenterol Clin North Am*. 2017;46:881893.

5. Labrecque N, Cermakian N. Circadian clocks in the immune system. *J. Biol. Rhythms*. 2015; 30:277-90.

6. Oh-oka K, Kono H, Ishimaru K, Miyake K, Kubota T, Ogawa H, et al. Expressions of tight junction proteins Occludin and Claudin-1 are under the circadian control in the mouse large

intestine: implications in intestinal permeability and susceptibility to colitis. PloS one. 2014;9: e98016.

7. Lange T, Dimitrov S, Born J. Effects of sleep and circadian rhythm on the human immune system. *Ann. N. Y. Acad. Sci*. 2010;1193:48-59.

8. Forsyth CB, Voigt RM, Burgess HJ, Swanson GR, Keshavarzian A. Circadian rhythms, alcohol and gut interactions. *Alcohol*. 2015;49:389-98.

9. Parkar SG, Kalsbeek A, Cheeseman JF. Potential role for the gut microbiota in modulating host circadian rhythms and metabolic health. *Microorganisms*. 2019;7:41.

10. Ruan W, Yuan X, Eltzschig HK. Circadian rhythm as a therapeutic target. *Nat. Rev. Drug Discov*. 2021;20:287-307.

11. Allada R, Bass J. Circadian mechanisms in medicine. *NEJM*. 2021;384:550-61.

12. Palmieri O, Mazzoccoli G, Bossa F, Maglietta R, Palumbo O, Ancona N, et al. Systematic analysis of circadian genes using genome-wide cDNA microarrays in the inflammatory bowel disease transcriptome. *Chronobiol. Int*. 201;32:903-16.

13. Mosna K, Janega P, Sedlak J, Babal P. Complex changes of circadian proteins expression in inflammatory bowel disease. *Bratislava Bratisl. Med. J*. 2021;122: 235-241.

14. Liu X, Yu R, Zhu L, Hou X, Zou K. Bidirectional regulation of circadian disturbance and inflammation in inflammatory bowel disease. *Inflamm. Bowel Dis*. 2017;23:1741-51.

15. Wang D, Yin H, Wang X, Wang Z, Han M, He Q, et al. Influence of sleep disruption on inflammatory bowel disease and changes in circadian rhythm genes. *Heliyon*. 2022;8:e11229.

16. Ditmer M, Gabryelska A, Turkiewicz S, Białasiewicz P, Małecka-Wojciesko E, Sochal M. Sleep problems in chronic inflammatory diseases: prevalence, treatment, and new perspectives: a narrative review. *J. Clin. Med*. 2021;11:67.

17. Sobolewska-Włodarczyk A, Włodarczyk M, Szemraj J, Stec-Michalska K, Fichna J, Wiśniewska-Jarosińska M. Circadian rhythm abnormalities–association with the course of inflammatory bowel disease. *Pharmacol. Rep*. 2016;68:847-51.

18. Voigt RM, Forsyth CB, Green SJ, Mutlu E, Engen P, Vitaterna MH, et al. Circadian disorganization alters intestinal microbiota. PloS one. 2014;9:e97500.

19. Gombert M, Carrasco-Luna J, Pin-Arboledas G, Codoñer-Franch P. The connection of circadian rhythm to inflammatory bowel disease. Translational Research. 2019;206:107-18.

20. Bechtold DA, Gibbs JE, Loudon AS. Circadian dysfunction in disease. *TIPS*. 2010;31:191-8.

21. Dufoo-Hurtado E, Wall-Medrano A, Campos-Vega R. Naturally-derived chronobiotics in chrononutrition. *Trends Food Sci Technol*. 2020;95:173-82.

22. Canani RB, Di Costanzo M, Leone L, Pedata M, Meli R, Calignano A. Potential beneficial effects of butyrate in intestinal and extraintestinal diseases. *WJG*. 2011;17:1519–1528.

23. Parada Venegas D, De la Fuente MK, Landskron G, González MJ, Quera R, Dijkstra G, et al. Short chain fatty acids (SCFAs)-mediated gut epithelial and immune regulation and its relevance for inflammatory bowel diseases. *Front. immunol*. 2019;10: 277.

24. Dou X, Gao N, Yan D, Shan A. Sodium butyrate alleviates mouse colitis by regulating gut microbiota dysbiosis. *Animals*. 2020;10:1154.

25. Segain JP, Raingeard de la Blétière D, Bourreille A, Leray V, Gervois N, Rosales C, et al. Butyrate inhibits inflammatory responses through NFkappaB inhibition: implications for Crohn's disease. *Gut*. 2000;47:397-403.

26. Macia L, Tan J, Vieira AT, Leach K, Stanley D, Luong S, et al. Metabolite-sensing receptors GPR43 and GPR109A facilitate dietary fibre-induced gut homeostasis through regulation of the inflammasome. *Nat. Commun*. 2015;6:1-15.

27. Silva JP, Navegantes-Lima KC, Oliveira AL, Rodrigues DV, Gaspar SL, Monteiro VV, et al. Protective mechanisms of butyrate on inflammatory bowel disease. *Curr. Pharm. Des.* 2018;24 :4154-66.

28. Morshedzadeh N, Shahrokh S, Aghdaei HA, Pourhoseingholi MA, Chaleshi V, Hekmatdoost A, et al. Effects of flaxseed and flaxseed oil supplement on serum levels of inflammatory markers, metabolic parameters and severity of disease in patients with ulcerative colitis. *Complement Ther Med*. 2019;46:36-43.

29. Roshanravan N, Mesri Alamdari N, Asghari Jafarabadi M, Mohammadi A and et.al. Effects of oral butyrate and inulin supplementation on inflammationinduced pyroptosis pathway in type 2 diabetes: A randomized, double-blind, placebo-controlled trial. *Cytokine*. 2020;131:155101.

30. Amiri, Hosseini SA, Roshanravan N, Saghafi-Asl M, Tootoonchian M. The effects of sodium butyrate supplementation on the expression levels of PGC-1α, PPARα, and UCP-1 genes, serum level of GLP-1, metabolic parameters, and anthropometric indices in obese individuals on weight loss diet: a study protocol for a triple-blind, randomized, placebo-controlled clinical trial. *Trials*. 2023;24:489.

31. Lewis JD, Chuai S, Nessel L, Lichtenstein GR, Aberra FN, Ellenberg JH. Use of the noninvasive components of the Mayo score to assess clinical response in ulcerative colitis. *Inflamm. Bowel Dis*. 2008;14:1660-6.

32. Farrahi Moghaddam J, Nakhaee N, Sheibani V, Garrusi B, Amirkafi A. Reliability and validity of the Persian version of the Pittsburgh Sleep Quality Index (PSQI-P). *Sleep Breath*. 2012;16:79-82.

33. Verissimo R. Quality of life in inflammatory bowel disease: psychometric evaluation of an IBDQ cross-culturally adapted version. 2008; 17:439-44.

34. Rustgi SD, Kayal M, Shah SC. Sex-based differences in inflammatory bowel diseases: a review. *Therap Adv Gastroenterol*. 2020; 13: 1756284820915043.

35. Prelipcean CC, Mihai C, Gogalniceanu P, Mihai B. What is the impact of age on adult patients with inflammatory bowel disease? *Clujul Med*. 2013; 86: 3–9.

36. Santhi N, Lazar AS, McCabe PJ, Lo JC, Groeger JA, Dijk DJ. Sex differences in the circadian regulation of sleep and waking cognition in humans. *Proc Natl Acad Sci U S A*. 2016; 113: 2730–2739.

37. Zhang C, Tait C, Minacapelli CD, Bhurwal A, Gupta K, Amin R, Rustgi VK. The Role of Race, Sex, and Age in Circadian Disruption and Metabolic Disorders. *Gastro Hep Advances*. 2022;1:471–479.

38. Pooja S, Salvi and Robert A. Cowles. Butyrate and the Intestinal Epithelium: Modulation of Proliferation and Inflammation in Homeostasis and Disease. *Cells*. 2021; 10: 1775.

39. Gibbs B, Brown BI. Butyrate Therapy for Treatment-Resistant Ulcerative Colitis: A Case Study. *Nutr Med J*. 2022;1:60-7.

40. www.bodybio.com

41. Leone V, Gibbons SM, Martinez K, Hutchison AL, Huang EY, Cham CM, et al. Effects of diurnal variation of gut microbes and high-fat feeding on host circadian clock function and metabolism. *Cell host & microbe*. 2015;17:681-9.

42. Tahara Y, Yamazaki M, Sukigara H, Motohashi H, Sasaki H, Miyakawa H, et al. Gut microbiota-derived short chain fatty acids induce circadian clock entrainment in mouse peripheral tissue. *Sci. Rep*. 2018;8:1395.

43. Woo JS, Min H-K, Choi J-W, Moon JH, Park M-J, Kwok S-K, et al. Short-chain fatty acid butyrate induces IL-10-producing B cells by regulating circadian-clock-related genes to ameliorate Sjögren's syndrome. *J. Autoimmun*. 2021;119:102611.

44. Fawad JA, Luzader DH, Hanson GF, Moutinho Jr TJ, McKinney CA, Mitchell PG, et al. Histone deacetylase inhibition by gut microbe-generated short-chain fatty acids entrains intestinal epithelial circadian rhythms. *Gastroenterology*. 2022;163:1377-90.

45. Cook TM, Gavini CK, Jesse J, Aubert G, Gornick E, Bonomo R, et al. Vagal neuron expression of the microbiota-derived metabolite receptor, free fatty acid receptor (FFAR3), is necessary for normal feeding behavior. *Mol. Metab*. 2021;54:101350.

46. Tahara Y, Otsuka M, Fuse Y, Hirao A, Shibata S. Refeeding after fasting elicits insulin-dependent regulation of Per2 and Rev-erbα with shifts in the liver clock. *J. Biol. Rhythms*. 2011;26:230-40.

47. Kon N, Hirota T, Kawamoto T, Kato Y, Tsubota T, Fukada Y. Activation of TGF-β/activin signalling resets the circadian clock through rapid induction of Dec1 transcripts. *Nat. Cell Biol*. 2008;10:1463-9.

48. Grosicki GJ, Riemann BL, Flatt AA, Valentino T, Lustgarten MS. Self-reported sleep quality is associated with gut microbiome composition in young, healthy individuals: a pilot study. *Sleep Medicine*. 2020;73:76-81.

49. Gao T, Wang Z, Dong Y, Cao J, Chen Y. Butyrate Ameliorates Insufficient Sleep-Induced Intestinal Mucosal Damage in Humans and Mice. *Microbiol. Spectr*. 2023;11:e02000-22.

50. Szentirmai É, Millican NS, Massie AR, Kapás L. Butyrate, a metabolite of intestinal bacteria, enhances sleep. *Sci. Rep*. 2019;9:7035.

51. Morshedzadeh N, Shahrokh S, Chaleshi V, Karimi S, Mirmiran P, Zali MR. The effects of flaxseed supplementation on gene expression and inflammation in ulcerative colitis patients: An open-labelled randomised controlled trial. *Int. J. Clin. Pract*. 2021;75:e14035.

52. Hegazy SK, El-Bedewy MM. Effect of probiotics on pro-inflammatory cytokines and NF-κB activation in ulcerative colitis. *WJG*. 2010;16: 4145-51.

53. Simeoli R, Mattace Raso G, Pirozzi C, Lama A, Santoro A, Russo R, et al. An orally administered butyrate-releasing derivative reduces neutrophil recruitment and inflammation in dextran sulphate sodium-induced murine colitis. *Br. J. Pharmacol*. 2017;174:1484-96.

54. Lührs H, Gerke T, Müller J, Melcher R, Schauber J, Boxberger F, et al. Butyrate inhibits NF-κB activation in lamina propria macrophages of patients with ulcerative colitis. *Scand. J. Gastroenterol*. 2002;37:458-66.

55. Vernia P, Annese V, Bresci G, d'Albasio G, D'Incà R, Giaccari S, et al. Topical butyrate improves efficacy of 5-ASA in refractory distal ulcerative colitis: results of a multicentre trial. *Eur J Clin Invest*. 2003;33:244-8.

56. Facchin S, Vitulo N, Calgaro M, Buda A, Romualdi C, Pohl D, et al. Microbiota changes induced by microencapsulated sodium butyrate in patients with inflammatory bowel disease. *Neurogastroenterol. Motil*. 2020;32:e13914.

57. Vernero M, De Blasio F, Ribaldone DG, Bugianesi E, Pellicano R, Saracco GM, et al. The usefulness of microencapsulated sodium butyrate add-on therapy in maintaining remission in patients with ulcerative colitis: a prospective observational study. *Clin. Med*. 2020;9:3941.

58. Caviglia GP, De Blasio F, Vernero M, Armandi A, Rosso C, Saracco GM, et al. Efficacy of a Preparation Based on Calcium Butyrate, Bifidobacterium bifidum, Bifidobacterium lactis, and Fructooligosaccharides in the Prevention of Relapse in Ulcerative Colitis: A Prospective Observational Study. *J. Clin. Med*. 2021;10:4961.

59. Sitkin S, Vakhitov T, Pokrotnieks J. How to increase the butyrate-producing capacity of the gut microbiome: do IBD patients really need butyrate replacement and butyrogenic therapy? *JCC* 2018;12:881-2.

60. Gonçalves P, Araújo JR, Di Santo JP. A cross-talk between microbiota-derived short-chain fatty acids and the host mucosal immune system regulates intestinal homeostasis and inflammatory bowel disease. *Inflamm. Bowel Dis*. 2018;24:558-72.

Revised-Manuscript.docx

ORIGINALITY REPORT

73%
SIMILARITY INDEX

PRIMARY SOURCES

1	www.researchsquare.com<br>Internet	5782 words — 66%
2	digital.library.adelaide.edu.au<br>Internet	65 words — 1%
3	Donya Firoozi, Seyed Jalil Masoumi, Seyed Mohammad Kazem Hosseini Asl, Aurélie Labbe et al. "The Effects of Short-Chain Fatty Acid-Butyrate Supplementation on the Expression of Circadian-Clock Genes and Sleep Quality in Patients with Active Ulcerative Colitis: A Double-Blind Randomized Controlled Trial", Research Square Platform LLC, 2023<br>Crossref Posted Content	52 words — 1%
4	ijns.sums.ac.ir<br>Internet	45 words — 1%
5	sciety.org<br>Internet	43 words — < 1%
6	trialsjournal.biomedcentral.com<br>Internet	33 words — < 1%
7	brieflands.com<br>Internet	32 words — < 1%
8	www.frontiersin.org	

Internet

28 words — < 1%

9 Sajjad Moradi, Mehdi Zobeiri, Awat Feizi, Cain C. T. Clark, Mohammad Hassan Entezari. "The Effects of Spirulina (Arthrospira platensis) Supplementation on Anthropometric Indices, Blood Pressure, Sleep Quality, Mental Health, Fatigue Status, and Quality of Life in Patients with Ulcerative Colitis: A Randomized, Double-blinded, Placebo-controlled Trial", International Journal of Clinical Practice, 2021
Crossref

27 words — < 1%

10 bodybio.com
Internet

25 words — < 1%

11 academic.oup.com
Internet

20 words — < 1%

12 pdfs.semanticscholar.org
Internet

15 words — < 1%

13 www.omicsdi.org
Internet

15 words — < 1%

14 bmcpregnancychildbirth.biomedcentral.com
Internet

13 words — < 1%

15 docplayer.net
Internet

13 words — < 1%

16 www.gmj.ir
Internet

13 words — < 1%

17 Maryam Samsami-kor, Naser Ebrahimi Daryani, Parisa Rezanejad Asl, Azita Hekmatdoost. "Anti-Inflammatory Effects of Resveratrol in Patients with Ulcerative

12 words — < 1%

Colitis: A Randomized, Double-Blind, Placebo-controlled Pilot Study", Archives of Medical Research, 2015
Crossref

18 Rozita Nasseri, Mojtaba Malek, Rokhsareh Aghili, Ameneh Ebrahim Valojerdi, Mohammad Ebrahim Khamseh. "Disturbed sleep in type 2 diabetes mellitus independent of chronic complications, pain, and nocturia", International Journal of Diabetes in Developing Countries, 2015
Crossref
12 words — < 1%

19 Nava Morshedzadeh, Shabnam Shahrokh, Vahid Chaleshi, Soheila Karimi, Parvin Mirmiran, Mohammad Reza Zali. "The Effects of Flaxseed Supplementation on Gene Expression and Inflammation in Ulcerative Colitis Patients: an open-labeled randomized controlled trial", International Journal of Clinical Practice, 2021
Crossref
11 words — < 1%

20 Asemi, Z, M Samimi, Z Tabassi, and A Esmaillzadeh. "The effect of DASH diet on pregnancy outcomes in gestational diabetes: a randomized controlled clinical trial", European Journal of Clinical Nutrition, 2014.
Crossref
10 words — < 1%

21 Sharif, Farkhondeh, Alireza Shoul, Mansour Janati, Javad Kojuri, and Najaf Zare. "The effect of cardiac rehabilitation on anxiety and depression in patients undergoing cardiac bypass graft surgery in Iran", BMC Cardiovascular Disorders, 2012.
Crossref
10 words — < 1%

22 ashpublications.org
Internet
10 words — < 1%

23 assets.researchsquare.com
Internet

10 words — < 1%

24 escholarship.org
Internet
10 words — < 1%

25 jyx.jyu.fi
Internet
10 words — < 1%

26 mafiadoc.com
Internet
10 words — < 1%

27 www.accessdata.fda.gov
Internet
10 words — < 1%

28 Arezoo Haseli, Farideh Egdampur, Kowsar Qaderi, Mohammad Reza Kaffashian, Ali Delpisheh. "Nurses and midwives' sleep quality and its associated factors during the early COVID-19 pandemic in Iran", Heliyon, 2023
Crossref
9 words — < 1%

29 Zeinab Khosravi, Amir Hadi, Helda Tutunchi, Mohammad Asghari-Jafarabadi et al. "The effects of butyrate supplementation on glycemic control, lipid profile, blood pressure, nitric oxide level and glutathione peroxidase activity in type 2 diabetic patients: A randomized triple -blind, placebo-controlled trial", Clinical Nutrition ESPEN, 2022
Crossref
9 words — < 1%

30 journals.lww.com
Internet
9 words — < 1%

31 www.journalslibrary.nihr.ac.uk
Internet
9 words — < 1%

32 Zhou, Yuyin. "Gut Microbial Metabolism of Aromatic Amino Acids Under Chemical and
8 words — < 1%

Microbiota Interventions", University of Minnesota, 2023
ProQuest

33	docksci.com<br>Internet	8 words — < 1%
34	www.mdpi.com<br>Internet	8 words — < 1%
35	www.science.gov<br>Internet	8 words — < 1%
36	Zachary Barrett-O'Keefe. "Maximal Strength Training: The Effects on Intramuscular Economy and its Determinants : 1345", Medicine & Science in Sports & Exercise, 05/2010<br>Crossref	7 words — < 1%
37	Mohammad Zamani, Mahlagha Nikbaf-Shandiz, Yasaman Aali, Niloufar Rasaei, Mahtab Zarei, Farideh Shiraseb, Omid Asbaghi. "The effects of acarbose treatment on cardiovascular risk factors in impaired glucose tolerance and diabetic patients: a systematic review and dose–response meta-analysis of randomized clinical trials", Frontiers in Nutrition, 2023<br>Crossref	6 words — < 1%
38	www.ncbi.nlm.nih.gov<br>Internet	6 words — < 1%

EXCLUDE QUOTES	OFF	EXCLUDE SOURCES	OFF
EXCLUDE BIBLIOGRAPHY	ON	EXCLUDE MATCHES	OFF
