# Supplementary material for: Effects of short-chain fatty acid-butyrate supplementation on expression of circadian-clock genes, sleep quality, and inflammation in patients with active ulcerative colitis: a double-blind randomized controlled trial
Source: Lipids Health Dis. 2024 Jul 13;23:216. doi: 10.1186/s12944-024-02203-z (PMC11245831; doi:10.1186/s12944-024-02203-z)
Supplement: Supplementary file 4 — Supplementary Material 4 [file 12944_2024_2203_MOESM4_ESM.pdf]

Dear Editor,

According to the overall similarity index checked by iThenticate, we have noted that the preprint of our manuscript is available online on ResearchSquare with the DOI doi.org/10.21203/rs.3.rs-3705016/v1. The software has identified a 67% similarity with our preprint of manuscript. Upon checking the primary sources of similarity referenced as number 1 and 3 this fact will be clear. By excluding these similarities, the final similarity is reduced to 6%.

Sincerely,

Seyed Jalil Masoumi, MD, Ph.D

Clinical Nutrition Department, School of Nutrition and Food Sciences, Shiraz University of Medical Sciences, Shiraz, Iran

Email: sjm@sums.av.ir, masoumi7415@gmail.com,

Phone: +98 917-315-026
